# ‘Right Now, Sophie ^∗^Swims in the Pool?!’: Brain Potentials of Grammatical Aspect Processing

**DOI:** 10.3389/fpsyg.2015.01764

**Published:** 2015-11-23

**Authors:** Monique Flecken, Kelly Walbert, Ton Dijkstra

**Affiliations:** ^1^Neurobiology of Language Department, Max Planck Institute for PsycholinguisticsNijmegen, Netherlands; ^2^Department of Psychology, University of ChicagoChicago, IL, USA; ^3^Donders Institute for Brain, Cognition and Behaviour, Centre for Cognition, Radboud UniversityNijmegen, Netherlands

**Keywords:** grammatical aspect, ERPs (event-related potentials), sentence processing, semantic processing, Early Negativity, morpho-syntactic processing, aspect processing

## Abstract

We investigated whether brain potentials of grammatical aspect processing resemble semantic or morpho-syntactic processing, or whether they instead are characterized by an entirely distinct pattern in the same individuals. We studied aspect from the perspective of *agreement* between the temporal information in the context (temporal adverbials, e.g., *Right now*) and a morpho-syntactic marker of grammatical aspect (e.g., progressive *is* swimm*ing*). Participants read questions providing a temporal context that was progressive (*What is Sophie doing in the pool right now?*) or habitual (*What does Sophie do in the pool every Monday?*). Following a lead-in sentence context such as *Right now, Sophie…*, we measured event-related brain potentials (ERPs) time-locked to verb phrases in four different conditions, e.g., (a) *is swimming* (control); (b) *^∗^is cooking* (semantic violation); (c) *^∗^are swimming* (morpho-syntactic violation); or (d)?*swims* (aspect mismatch); …*in the pool.*” The collected ERPs show typical N400 and P600 effects for semantics and morpho-syntax, while aspect processing elicited an Early Negativity (250–350 ms). The aspect-related Negativity was short-lived and had a central scalp distribution with an anterior onset. This differentiates it not only from the semantic N400 effect, but also from the typical LAN (Left Anterior Negativity), that is frequently reported for various types of agreement processing. Moreover, aspect processing did not show a clear P600 modulation. We argue that the specific context for each item in this experiment provided a trigger for agreement checking with temporal information encoded on the verb, i.e., morphological aspect marking. The aspect-related Negativity obtained for aspect agreement mismatches reflects a violated expectation concerning verbal inflection (in the example above, the expected verb phrase was *Sophie is X-ing* rather than *Sophie X-s* in condition d). The absence of an additional P600 for aspect processing suggests that the mismatch did not require additional reintegration or processing costs. This is consistent with participants’ *post hoc* grammaticality judgements of the same sentences, which overall show a high acceptability of aspect mismatch sentences.

## Introduction

When we tell others about events or actions taking place, we usually express not only where they happened and to whom, but also *when* they occurred. In finite sentences describing events or actions, grammar may in fact require speakers to make explicit distinctions with respect to the temporal properties of events. For instance, speakers may need to use grammatical markers of tense to indicate whether the event took place in the past or is taking place in the present. In some languages, speakers must specify whether an event has just begun, is in progression, has reached a state of completion, or involves an instance of a recurring action, for example. The temporal contours of an event are marked by what we call *grammatical aspect*. In some languages (e.g., Russian, English) but not in others (German, Swedish), grammatical aspect involves morphological marking on the main lexical verb in a sentence through affixes or periphrastic constructions, e.g., progressive aspect in English: *he is cooking.*

Aspect marking is relevant for a listener’s conception of the temporal contours (i.e., boundaries, inner phases, duration) of the situation described, and thus contributes substantially to sentence meaning (for empirical evidence, see e.g., Anderson et al., 2013; Flecken and Gerwien, 2013; Parrill et al., 2013; Zhou et al., 2014). The progressive highlights the intermediate phases of an event and refers to a specific ‘ongoing’ instance (*he is cooking*), rather than making a generic statement or referring to a recurring event which is expressed using a verb unmarked for aspect in English (*he cooks*, meaning he is a chef; Comrie, 1976; Dahl, 2000). Importantly, grammatical aspect has scope over multiple sentences or a wider stretch of discourse. The extent to which an aspect marker can be considered ‘appropriate’ or ‘correct’ depends on semantic constraints given by the temporal frame of reference as specified by preceding context; an aspectually marked verb should be in temporal agreement with this frame of reference. For example, in English the answer to the question *What is John doing in the kitchen at the moment?* should contain a marker of progressive aspect: *At the moment, John is cooking in the kitchen*. The sentence *At the moment, John cooks in the kitchen* would be inappropriate due to the mismatch between the temporal context of a specific event currently in progress, created by the question, and the unmarked verb form *cooks*, which has a different temporal interpretation.

Given that grammatical aspect thus provides an interface between semantic and purely morpho-syntactic features of a sentence and serves to establish temporal-aspectual *agreement* with the preceding context, the question arises as to how temporal-aspectual information is processed, as reflected in event-related brain potentials (ERPs). Do ERPs of aspect processing resemble those typically associated with semantic processing (N400; cf. Kutas and Hillyard, 1980; Kutas and Federmeier, 2011) and/or those associated with morpho-syntactic processing (P600; cf. Osterhout and Holcomb, 1992; Hagoort et al., 1993; Coulson et al., 1998)? Or is there some resemblance to the electrophysiological pattern obtained specifically for grammatical agreement processing, i.e., a biphasic Left Anterior Negativity (LAN; negative peak roughly between 200 and 500 ms) followed by a P600 response? The latter pattern has been reported for grammatical agreement of person, gender, and number features between, e.g., an article and a noun, or a subject and a verb (see, e.g., Roehm et al., 2005; Mancini et al., 2011; review in Molinaro et al., 2011).

To date, little is known about ERPs of aspect agreement. Few studies have investigated grammatical aspect from the perspective of agreement between context and the morpho-syntactic marking of aspect on a verb. Previous investigations of grammatical aspect processing concerned verb forms that were locally erroneous with respect to the morpho-syntactic formation of grammatical aspect, for example, cases that constituted a conflict between two forms within the same verb phrase. The same holds for verb tense, a category that functions similar to aspect. For example, Osterhout and Nicol (1999) investigated the processing of ungrammatical sentences during reading such as *The cat’s won’t ^∗^eating (correct: eat) the food that Mary leaves for them*, which were claimed to be tense violations. The violation on the verb here is unrelated to the domain of time or temporality (given that there is no previously established temporal frame of reference), but rather the violation represents a local mismatch between the modal verb and the form of the lexical verb. Similarly, Allen et al. (2003) tested violations such as *The man will ^∗^worked (correct: work) on the platform*. In their study, the sentences that people read contained morpho-syntactic violations (conflict between modal *will* and form of the lexical verb *work*) that do not involve violations of tense agreement, as the verbal inflections did not mismatch with a preceding temporal context.

Steinhauer and Ullman (2002) examined true violations of temporality. Here, participants were presented with regular and irregular past tense forms preceded by a mismatching temporal adverb, thus creating a temporal mismatch. Participants read sentences like *Yesterday, I sailed Diane’s boat to Boston* (correct); *Yesterday, I ^∗^sail Diane’s boat to Boston* (tense violation) and *Yesterday, we ate Peter’s cake in the kitchen* (correct); *Yesterday, we ^∗^eat Peter’s cake in the kitchen* (tense violation). The violations triggered a LAN (a negativity with left anterior distribution) in the 300–500 ms time window, followed by a P600 (600–900 ms). More specifically, a centro-parietal N400 occurred in the first half of the early time window (300–400 ms) and a LAN effect was found in the second half (400–500 ms). The authors suggested that the centro-parietal negativities reflect differences in morpho-phonology of the regular and irregular verbs (i.e., *sail/sailed* versus *eat/ate)*. In all, the information provided by the temporal adverb was already used by the processing system as early as 300 or 400 ms after stimulus onset.

In a similar vein, Baggio (2008) investigated tense violations in Dutch. Participants read sentences that were again semantically shaped with respect to temporality by a preceding temporal adverb, as in *Afgelopen zondag lakte Vincent de kozijnen van zijn landhuis* (‘Last Sunday Vincent painted the window frames of his country house,’ correct) and *Afgelopen zondag ^∗^lakt Vincent de kozijnen van zijn landhuis* (‘Last Sunday Vincent *^∗^*paints the window frames of his country house,’ tense violation). A LAN was found (200–400 ms), followed by a P600 concentrated in the right posterior region. A relative negativity was also obtained in the 400–700 ms window at the sentence-final word. Referred to as a sentence-final negativity, this effect resembled an N400 in terms of scalp distribution but it had a later peak and was more prolonged than a typical N400.

The most direct evidence regarding aspect processing comes from Zhang and Zhang (2008), an investigation of the ERP pattern of reading aspect violations in Mandarin Chinese. However, the type of violation again represented an ungrammaticality within a verb phrase: to signify perfective aspect in Chinese, the marker *yijing* appears before the verb and *le* appears after the verb. Similarly, *zhengzai* appears before the verb and *zhe* occurs after it to denote progressive aspect. To create an aspect violation, Zhang and Zhang used one perfective and one progressive marker in the same sentence: *Su Jun zhengzai (progressive) prepare (INF) le (perfective) fruit and cookies* [‘Su jun prepare (progr + perf. marker) fruit and cookies’]. Different responses were observed to semantic violations (other experimental condition) and the aspect violations. While the semantic violations elicited a classic N400 effect, the aspect violations triggered a negativity in the 200–400 ms time window, followed by a P600. The Early Negativity was strongest in the posterior and left central regions, differentiating it from a LAN in terms of scalp distribution. The authors suggested that this early negativity could be due to, either the detection of a closed-class “intruder” perfective marker, or to a failure to bind the progressive and perfective markers. They interpreted the subsequent P600 as a reflection of “syntactic repair or the monitoring and resolution of conflict caused by the aspect disagreement” (Zhang and Zhang, 2008, p. 1042).

A biphasic pattern consisting of an Early Negativity, often with a clear left anterior distribution (labeled the LAN) with a varied onset latency of 200–350 ms lasting until about 500 ms, followed by a P600, is frequently reported in the ERP literature on grammatical agreement processing. Agreement between a specific property of a linguistic element (semantic or morphological) and a morphological property of another element has been manipulated for grammatical number, person and gender, contrasting agreement with disagreement (violation) conditions (e.g., Osterhout and Mobley, 1995; Coulson et al., 1998; Hinojosa et al., 2003; Barber and Carreiras, 2005; Roehm et al., 2005; Molinaro et al., 2008; Mancini et al., 2011; for a review, see Molinaro et al., 2011). The biphasic LAN–P600 pattern is explained as representing an early stage during which there is a violation of the expected morpho-syntactic feature or form given the context (rendering the LAN), followed by a stage in which the processor structurally integrates the form with the context (P600). It has been argued that the specifics of the LAN depend on the type of agreement feature and constituent involved. For example, Molinaro et al. (2008) compared determiner-noun agreement on the basis of phonotactics (Italian masculine determiner *il/lo* alternation) and gender agreement rules (during reading): *The old woman with the [masculine LO (correct)/IL (phonotactic violation) or feminine LA (gender violation)] shawl (masculine)*. Agreement violation conditions elicited a LAN/Early Negativity (300–450 ms), followed by a P600. Comparing phonotactic to gender disagreement rendered subtle differences in the LAN/Early Negativity realm: gender agreement violations showed a more widespread and central scalp distribution (more N400-like) and phonotactic agreement violations displayed a clearer left anterior pattern. The authors argued that the extent to which lexical information plays a role in morpho-syntactic agreement computation affects the LAN. Specifically, a stronger reliance on lexical information increases the LAN’s resemblance to an N400 in terms of scalp topography and potentially also latency. There were also further differences in the P600 realm, with gender violations showing an enhanced positivity, that are possibly indicative of deeper reanalysis of gender agreement violations compared to phonotactics.

### The Present Study

To our knowledge, the present study is the first to directly investigate brain processing of aspect agreement between temporal context and verb morphology, filling a gap in ERP research on sentence processing. We compared aspect processing (in terms of an aspect agreement mismatch: *Right now, Sophie ^∗^swims in the pool/ Every day, Sophie ^∗^is swimming in the pool*) to semantic processing (semantic violation: *Right now, Sophie ^∗^is cooking in the pool)* and to number agreement processing (morpho-syntactic violation: *Right now, Sophie ^∗^are swimming in the pool)* in one and the same group of English-speaking participants. The design thus allowed a direct comparison between semantic, morpho-syntactic and aspect processing. We predicted that semantic violations would elicit a typical central-posterior N400 effect (cf. Kutas and Hillyard, 1980), whereas number agreement violations would result in a P600 effect (Osterhout and Mobley, 1995; Hagoort and Brown, 2000; Kaan, 2002), potentially preceded by a LAN.

Specific ERP evidence on aspect agreement processing is lacking, but we expected (one or some of) the following conditional modulations of different ERP components to occur for aspect agreement mismatches: if the processing of aspect agreement mismatches resembles processing of semantic anomalies, an N400 modulation should be observed. If, however, processing of aspect mismatches resembles processing of morpho-syntactic violations, a P600 modulation should be observed. Finally, instead of or in addition to N400 and P600 effects, a LAN/Early Negativity might be obtained as early as 200–400 ms, in line with studies on grammatical agreement processing and the evidence regarding tense agreement (a category functionally similar to aspect) specifically (Steinhauer and Ullman, 2002; Baggio, 2008).

In the first part of our study, native speakers of English performed a sentence-reading task while EEG was recorded. Items were presented as question-answer pairs. A question related to the activity of a specific person at a specific location and time was presented first (e.g., *What is Sophie doing in the pool, right now?*), followed by an answer (*Right now, Sophie is swimming in the pool*). Answer sentences contained a verb whose form was manipulated to match each of the four conditions outlined above: control condition (correct: *is swimming*), semantic violation (^∗^*is cooking*), morpho-syntactic violation (*^∗^are swimming*), aspect mismatch (*^∗^swims*). The aspect mismatch sentences consisted of two types, given that in English, the progressive contrasts with verbs unmarked for aspect, which are interpreted as describing habitual or generic information (Comrie, 1976; see materials below).

In the second part of our study, the same participants completed an English proficiency test to ensure high native language proficiency, and two overt acceptability judgment tasks. In the first task, participants judged the typicality of verb-location prepositional phrase (PP) pairings in sentences from the control condition (e.g., *swim in the pool*) and the semantic violation (e.g., *cook in the pool*) condition. In the second task, participants rated the grammaticality of sentences from the control condition, morpho-syntactic violation condition, and aspect mismatch condition. The judgment tasks assessed how participants overtly judged aspect mismatches relative to the other two violation types and controls.

## Materials and Methods

### Participants

Thirty right-handed participants (mean age 24.8 years, *SD* = 4.0; 9 males) took part in the study for payment. All participants had normal or corrected-to-normal vision, and none reported neurological or psychological disorders. All participants gave written consent to take part in the experiment, which was approved by the ethics committee of the Faculty of Social Sciences at Radboud University Nijmegen (ECG2012-2711-059). Participants came from the United States, Canada, the United Kingdom and Ireland, and were temporarily residing in Nijmegen at the time of testing (time of residence between 1 month and 7 years; average 1.2 years). Most participants were either (1) exchange students spending 1 or 2 semesters studying in the Netherlands and taking English-taught classes, or (2) international students studying in a 1- or 2-year English-taught Master’s program. One participant had lived in the Netherlands for 7 years. All participants reported having no or only very little knowledge of Dutch. English was the language they used almost exclusively in their daily lives.

### Materials

The materials for the reading task consisted of 160 question-answer pairs and 80 comprehension question filler trials for a total of 240 trials per participants. There were 20 unique items (verb-location pairs) that were used as the basis for all question-answer pairs (see the Appendix). These items were pretested in a typicality rating task on proficient Dutch L2 users of English (*N* = 20) prior to the experiment, to ensure that they were viewed as semantically correct and highly typical (e.g., *swim in the pool*), whereas their counterparts (mismatching verb-location pairs, *cook in the pool*) in the semantic violation condition were viewed as atypical. Native English participants that took part in this study also performed this rating task after the EEG experiment. Question-answer pairs began with a question that asked about the activities of a specific person at a specific location and that also set up one of two possible temporal contexts, an ongoing ‘progressive’ event context (*What is Sophie doing in the pool today*?) or a habitual event context (*What does Sophie do in the pool every Saturday*?). Temporal context was explicitly marked by either a temporal adverbial expressing ongoingness (e.g., *right now, at the moment*) or a temporal adverbial expressing habitual, generic, or repetitive action (e.g., *every Saturday, every weekend, every holiday*).

There were four answer types: answers that contained no violation (control condition), answers containing a number agreement violation (morpho-syntactic violation condition), answers containing a semantic anomaly (semantic violation condition), and answers containing an aspect agreement violation (aspect mismatch condition). To construct the morpho-syntactic violation and aspect mismatch items, the verb form in the answer sentences was manipulated. To construct the semantic violation items, a semantically anomalous verb was paired with the location. Items were spliced, creating two temporal contexts based on the critical verb form: ongoing progressive context (verb phrase [VP] type *long*, owing to the presence of the copula ‘is/are’) and habitual ‘simple present’ context (VP type *short*). **Table 1** shows an overview of all conditions.

**Table 1:** Examples of sentences in each condition and VP type (item *swim in the pool*).

Condition	VP type	Example of preceding sentence	Example of critical sentence
Control	Long	What is Sophie doing in the pool today?	Today, Sophie is swimming in the pool
	Short	What does Sophie do in the pool every Monday?	Every Monday, Sophie swims in the pool
Semantic violation	Long	What is the boy doing in the pool today?	Today, the boy is cooking in the pool
	Short	What does the boy do in the pool every Monday?	Every Monday, the boy cooks in the pool
Morpho-syntactic	Long	What is the woman doing in the pool right now?	Right now, the woman are swimming in the pool
Violation	Short	What does the woman do in the pool every Tuesday?	Every Tuesday, the woman swim in the pool
Aspect mismatch	Long	What does John do in the pool every Tuesday?	Every Tuesday, John is swimming in the pool
	Short	What is John doing in the pool right now?	Right now, John swims in the pool

*Critical verb phrase used for ERP timelocking is underlined.*

Ten pseudorandomized lists consisting of 10 blocks of 24 trials (20 question-answer pairs and 4 filler trials with comprehension questions) were created using a Latin square design. The order of the conditions between blocks for each item was varied. Each item occurred once in each block, and each block had an even split of items from each VP type. Items sharing both condition and VP type were never presented in a row. To ensure that participants paid attention throughout the experiment, and also to increase the overall number of non-anomalous sentences in the experiment, 80 additional filler question-answer pairs were distributed in a pseudorandom order that was fixed across all lists. Fillers consisted of a question/answer pair resembling a test item, followed by a simple comprehension question that required a yes/no answer from the participant (by button press) and referred to information presented in the immediately previous filler question-answer pair (e.g., *Is Gina in the city right now?*).

### Procedure

Participants were tested individually in a soundproof booth. Each participant was seated in a comfortable chair in front of a computer screen at a viewing distance of 100 cm. Participants were instructed by a native speaker of English that they would see question-answer pairs presented on the screen, with questions presented in full and answers presented in chunks. Each question was preceded by a fixation cross (2000 ms) and a blank screen (350 ms), and followed by another fixation cross (1350 ms). Questions remained on the screen for 3000 ms; for the subsequent answer sentence, single words (including the verb phrase) were presented for 350 ms, with an ISI of 350 ms (the temporal adverbial phrase at the start of the answer sentence was presented for 500 ms). Participants were informed that they would occasionally have to answer a question referring to the previous question-answer pair, by pressing “yes” or “no” on the button box placed in front of them. Participants were asked to blink only when a full question or a fixation cross was presented on the screen.

In total, 240 trials were presented in five parts, each of which took about 10 min and was followed by a short self-timed break. Words were presented against a white background in 24-point size (Arial) in a centered position on a 19-inch CRT monitor, with a Neurobehavioral systems^TM^ Presentation script. Participants initially performed 10 practice trials. Filler questions referring to the previous filler trial were shown in full (i.e., *Does Lily exercise at the gym every Thursday*?). After participants had answered by pressing a button, the next trial was initiated.

After completing the reading task, participants completed a test of English proficiency known as LexTALE (Lemhöfer and Broersma, 2012). LexTALE is a lexical decision task, requiring a response of “yes” or “no” in relation to English words or non-words. Scores are computed on the basis of the number of correct/incorrect responses to words and non-words. In addition, participants took part in two tasks in which they rated sentences they had encountered earlier during the reading task. In the first task, participants rated the typicality of 40 test sentences (20 control, 20 semantic violation) on a scale of 1 to 5, based on how typical they considered the combination of the verb phrase (activity) with the subsequent PP (location; e.g., *swim in the kitchen*). As temporality was not relevant to the interpretation of semantic violations, sentences were presented without the adverb. In the second task, participants rated 120 sentences (40 control, 40 morpho-syntactic violation, 40 aspect mismatch condition) on a scale of 1 to 5, based on their perceived grammaticality of the sentences. The procedure took 2 h per participant.

### Data Preprocessing and Analyses

EEG was recorded from 27 cap-mounted Ag/AgCl electrodes (ActiCAP, Brainproducts). Five electrodes were placed on the midline sites Fz, FCz, Cz, Pz, and Oz. Eleven pairs were placed over the lateral sites F7/F8, F3/F4, FC5/FC6, FC1/FC2, T7/T8, C3/C4, CP5/CP6, CP1/CP2, P7/P8, P3/P4, and O1/O2. Horizontal eye movements were monitored by two additional electrodes placed at the outer left and right canthi. Vertical eye movements were monitored using two additional electrodes placed above and below the left eye. In addition, electrodes were placed on the left and right mastoid bones. During EEG recording, all electrodes were referenced to the left mastoid. All impedances were kept below 10 kΩ. Signals were recorded with a BrainAmp amplifier system, using a 150 Hz low-pass filter, a time constant of 10 s (0.016 Hz), and a 500 Hz sampling rate. The software package Brain Vision Analyzer 2^TM^ (Brain Products GmbH) was used to analyze the waveforms.

The EEG signal was re-referenced oﬄine to the mean of the right and left mastoid. EEG activity was filtered oﬄine with a bandpass zero phase shift filter (high cutoff: 30 Hz, 12 dB/oct). Eye blinks were mathematically corrected based on the automatic ocular correction procedure, implemented in BrainVision Analyzer 2^TM^. ERPs were timelocked to the presentation of the critical verb phrase in the answer sentence. Individual ERPs were computed from epochs ranging from -100 to 1000 ms after the onset of the critical verb phrase in the answer sentence and baseline corrected in reference to 100 ms of pre-stimulus activity. Segments (*N* = 40 per condition) were screened for artifacts based on a ±75 μV criterion; segments containing such artifacts were rejected, with no asymmetry over conditions. The remaining segments were averaged per participant and per condition. One participant was excluded from the analysis due to an excessive number of artifacts in the EEG signal (exclusion criterion: >25% rejected segments in at least one condition).

For the selected time windows of 250–350 ms (Early Negativity/LAN time window, see below), 350–500 ms (N400 time window) and 500–800 ms (P600 window), difference waves were computed by subtracting the control (correct) condition from each mismatch/violation condition [Aspect mismatch-correct (Aspect), Semantic violation-correct (Semantics), and Morpho-Syntactic violation-correct (Morpho-syntax)]. The resulting difference scores were then statistically analyzed using repeated measures analyses of variance (ANOVAs) with the following factors: For Lateral electrodes, Hemisphere (Left, Right), Anteriority (Anterior, Posterior), and Condition (Aspect, Semantics, Morpho-syntax). The Midline electrodes were analyzed separately in repeated measures ANOVAs with the factors Condition and Electrode (Fz, FCz, Cz, Pz, Oz).

**FIGURE 1:**
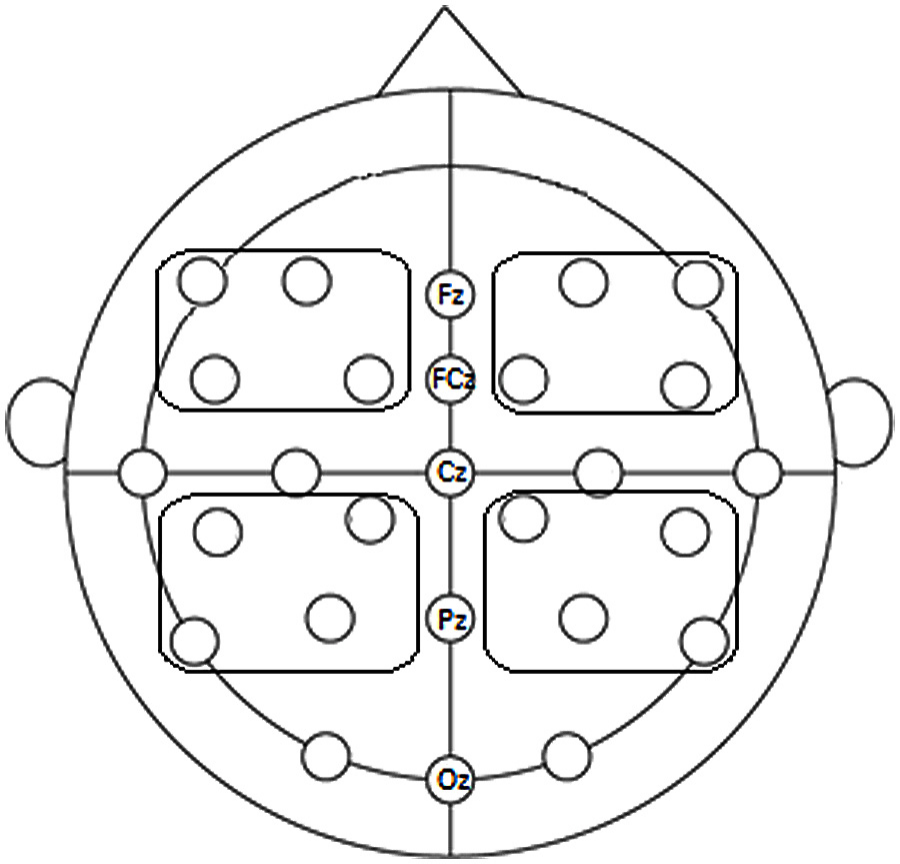
**EEG cap with five clusters of electrodes (Left Anterior, Right Anterior, Left Posterior, Right Posterior, and Midline electrodes)**.

We clustered the electrodes in five regions to reduce overall variance and the number of levels for follow-up analyses. Moreover, we were interested in the global topography (hemisphere, anteriority, and region) of potential effects on lateral electrodes – in particular the aspect effect’s resemblance to N400, P600, or LAN topography – and not so much in effects at single electrode sites (following, e.g., Roehm et al., 2005; Molinaro et al., 2008). There were four lateral regions (Left Anterior: mean activity of F7, F3, FC5, FC1; Right Anterior: mean activity of F4, F8, FC2, FC6; Left Posterior: mean activity of CP5, CP1, P7, P3; Right Posterior: mean activity of CP2, CP6, P4, P8) and one Midline region (Fz, FCz, Cz, Pz, Oz; **Figure 1**).

For lateral electrodes, when there was a significant or near-significant three-way interaction of the spatial factors Hemisphere and Anteriority with the Condition factor, repeated measures ANOVAs for each Region were conducted separately, with *post hoc* comparisons between the Aspect mismatch condition and each of the two other conditions (Bonferroni corrections were applied). On the basis of previous literature, aspect processing was hypothesized to elicit an effect with a specific scalp topography in the Early Negativity time window, i.e., a LAN (cf. Molinaro et al., 2011) or a more central-posteriorly distributed Early Negativity (Zhang and Zhang, 2008). Thus, to shed more light on the topography of aspect processing and its resemblance to LAN-like effects, the above mentioned planned comparisons between Aspect and Semantics, and Aspect and Morphosyntax were conducted for each of the above defined Regions on the scalp. For the Midline region, a significant interaction of Condition with the spatial factor Electrode was followed up by repeated measures analyses for each Electrode separately. Spatial factors are only reported when there was a (near-) significant interaction with the Condition factor. Overall, p-values were Greenhouse-Geisser corrected when the analysis involved more than one degree of freedom (original degrees of freedom are reported below). *P*-values are referred to as ‘ns’ when *p* > 0.09, and as a trend when they are between *p* = 0.05 and *p* = 0.08.

**FIGURE 2:**
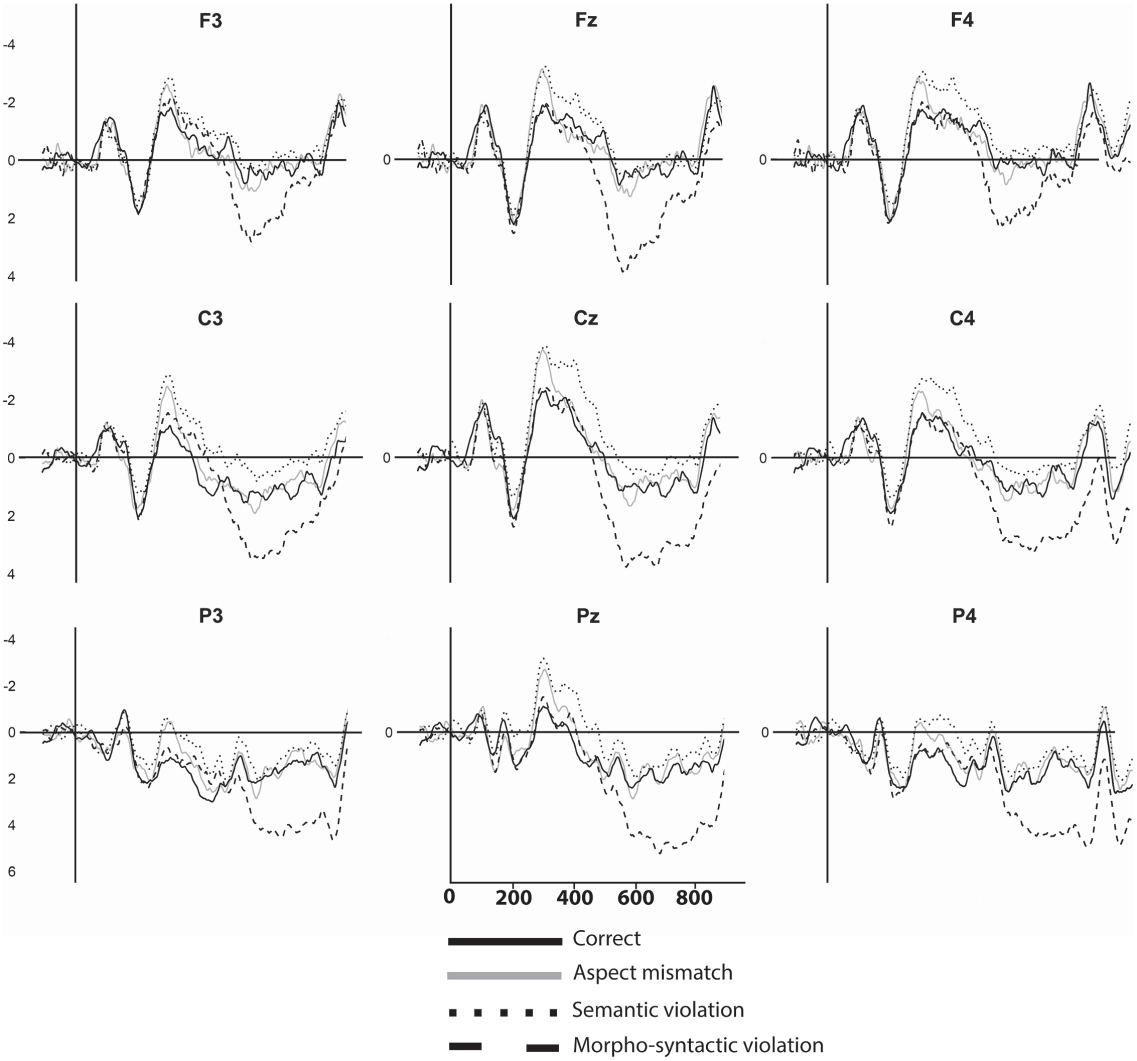
**Grand-averaged ERP waveforms (*N* = 29) on nine electrodes for four conditions, timelocked to the presentation of the verb phrase.** Negativity is plotted upward.

Time windows included a classic N400 time window (350–500 ms) typically associated with semantic processing (cf. Kutas and Hillyard, 1980) and the (early) P600 time window (500–800 ms) for morpho-syntactic processing (cf. Osterhout and Holcomb, 1992; Hagoort et al., 1993). An additional early time window of 250–350 ms was included, based on previous studies of tense processing (Baggio, 2008) and grammatical agreement processing (Molinaro et al., 2011), as well as visual inspection of the waveforms (**Figure 2** below). Preliminary analyses were conducted to check for potential differences between verb phrases of different lengths (long vs. short, e.g., *is swimming* vs. *swims*) in all time windows of interest.

## Results

### Task Performance

Task performance was assessed by the number of correct button presses in response to the 40 comprehension questions. On average, participants answered 94.10% of questions correctly, indicating that they paid attention to the task and were reading sentences for content.

### ERP Data

#### Control Analysis: Comparing Verb Phrase (VP) Length

Two different types of verb phrases were used in each condition, i.e., *swims* (average number of characters: 5.36) and *is swimming* (average number of characters: 9.68), each of which was presented in full on the screen. When participants viewed the critical verb phrase in one-word VP sentences (VP short, *swims*), they were immediately presented with a meaningful character, informative with respect to the type of activity described. In two-word VP sentences (VP long, *is swimming*), they were first confronted with the copula, informing them about aspect but not containing semantic information. Differences in VP length could potentially lead to processing differences, regardless of our experimental manipulations.

Therefore, we carried out preliminary analyses on average voltage activity on all electrodes for each time window of interest, testing for potential main effects of VP length (two levels: short, long), or an interaction of this factor with Condition (four levels: correct, aspect mismatch, semantic violation, morpho-syntactic violation). In the **350–500 ms** time window, the analysis rendered a significant Condition main effect [*F*(3,84) = 3.641, *p* < 0.05], but no main effect of VP length [*F*(1,28) = 0.272, *p* = 0.606, ns], and no VP length by Condition interaction [*F*(3,84) = 1.235, *p* = 0.302, ns]. In the **500–800 ms** time window, there was no main effect of VP length [*F*(1,28) = 1.611, *p* = 0.215, ns], a significant Condition main effect [*F*(3,84) = 10.617, *p* < 0.001], and once again no Condition by VP length interaction [*F*(3,84) = 0.421, *p* = 0.739, ns]. In the **250–350 ms** time window, a repeated measures ANOVA of VP length (2) by Condition (4) showed a main effect of Condition [*F*(3,84) = 5.182, *p* < 0.05], no effect of VP length [*F*(1,28) = 1.146, *p* = 0.293, ns], and no VP length by Condition interaction [*F*(3,84) = 1.357, *p* = 0.262, ns].

In the following analyses we pooled trials with shorter and longer verb phrases in each condition.

#### Condition Analyses

**Figure 2** shows grand averaged ERP waveforms for all four conditions, on nine frontal-to-parietal electrodes (F3, Fz, F4, C3, Cz, C4, P3, Pz, P4).

**Figure 2** above shows a steep short-lived negative peak for the aspect mismatch condition around 300 ms, clearest on frontal and central electrodes. The semantic violation condition displays a similar negative peak, which continues as a somewhat shallower central-posterior negativity lasting until around 500 ms, and which can be identified as an N400. The morpho-syntactic violation shows a large positive wave starting around 500 ms and is visible on nearly all electrodes (central-posterior focus) that can be identified as a P600. After 800 ms, the waveforms display a negative peak followed by a small positivity. In the present paper, we will only focus on the Early Negativity, the N400, and the early P600.

##### N400 time window (350–500 ms)

**FIGURE 3:**
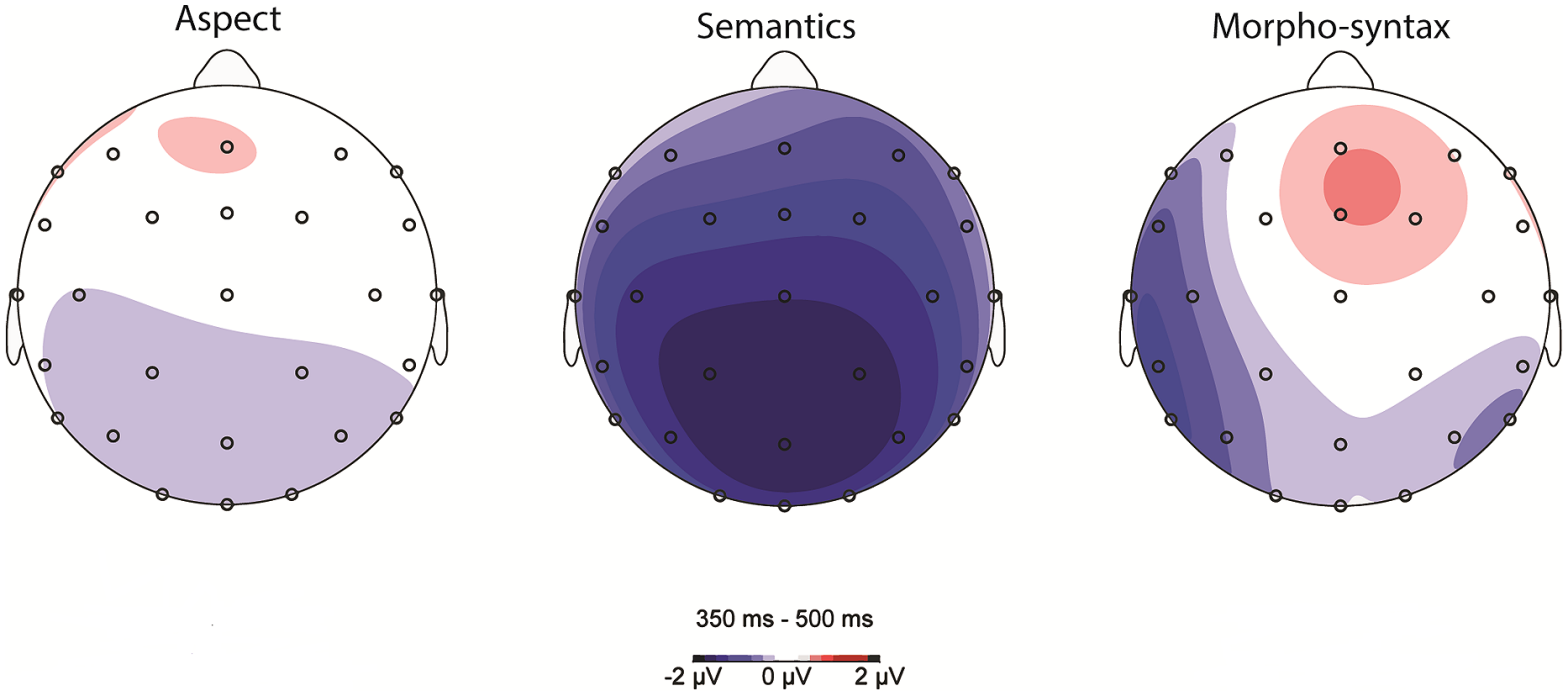
**Scalp topography of aspect, semantic, and morpho-syntactic processing (violation condition minus control condition) between 350 and 500 ms**.

**Figure 3** shows the scalp distribution of aspect, semantic, and morpho-syntactic processing (subtracting the control condition from each critical condition) in the N400 time window.

For the lateral electrodes, a Condition (3) by Anteriority (2) and Hemisphere (2) repeated measures ANOVA showed a trend for a main effect of Condition [*F*(2,56) = 2.903, *p* = 0.063], and a significant Condition by Hemisphere interaction [*F*(2,56) = 6.496, *p* < 0.05]. To target the topography of differences between the Aspect mismatch condition and each of the other two conditions, we looked at each Hemisphere separately. On the left hemisphere, there was a significant difference between the Aspect mismatch and the Semantic violation condition [*F*(1,28) = 7.00, *p* < 0.05], but there was no difference between Aspect and Morpho-syntax [*F*(1,28) = 1.516, *p* = 0.228, ns]. On the right hemisphere, the Aspect condition was again less negative than the Semantic condition [*F*(1,28) = 9.153, *p* < 0.05], but Aspect did not differ from the Morpho-syntactic violation condition [*F*(1,28) = 0.004, *p* = 0.949, ns). Overall, the Semantic condition showed more negative N400 amplitudes on both hemispheres, whereas Aspect and Morpho-syntax showed similarly small negativities; the factor Hemisphere did not play a role in our planned condition comparisons.

On midline electrodes, A Condition (3) by Electrode (5) analysis showed a main effect of Condition [*F*(2,56) = 5.831, *p* < 0.05], but no interaction of Condition with Electrode [*F*(8,224) = 1.154, *p* = 0.335, ns]. The Aspect mismatch condition differed from the Semantic violation condition (*p* < 0.05), but not from Morpho-syntactic violations (*p* = 0.99), with Semantic violations showing a stronger negativity.

Overall, the processing of aspect mismatches displayed a smaller negativity compared to semantic violations in the N400 time window; the latter condition showed the typical widespread N400 effect associated with semantic processing and the aspect condition differed significantly from the semantic condition on both lateral and midline electrodes. Similar to the processing of morpho-syntactic violations, aspect mismatches did not show the semantic N400 (no difference between aspect and morpho-syntax).

##### P600 time window (500–800 ms)

**FIGURE 4:**
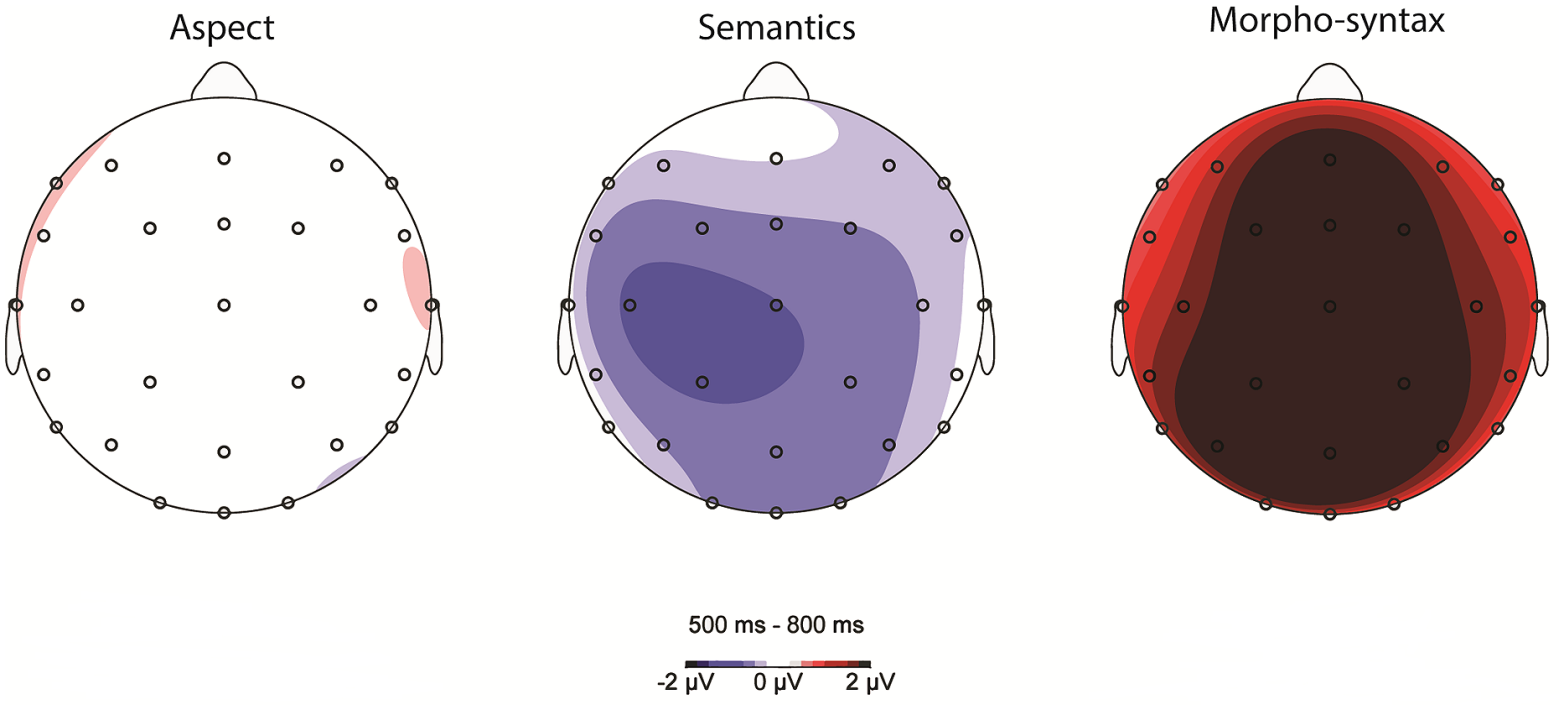
**Scalp topography of aspect, semantic, and morpho-syntactic processing (violation condition minus control condition) between 500 and 800 ms**.

**Figure 4** shows the scalp distribution of aspect, semantic, and morpho-syntactic processing (subtracting the control condition from each critical condition) in the 500–800 ms time window.

For the lateral electrodes, a Condition (3) by Anteriority (2) and Hemisphere (2) repeated measures ANOVA showed a Condition main effect [*F*(2,56) = 11.346, *p* < 0.001] and a significant three-way interaction of Condition, Anteriority, and Hemisphere [*F*(2,56) = 4.236, *p* < 0.05]. To explore this interaction, separate Condition analyses per Region were conducted. In the Left Anterior region, there was a Condition effect [*F*(2,56) = 12.676, *p* < 0.001]: the Aspect mismatch condition differed from Semantic (*p* < 0.05) as well as Morpho-syntactic processing (*p* < 0.05), in being more positive than the Semantic condition and more negative than the Morpho-syntactic condition. In the Right Anterior region, the pattern was similar [*F*(2,56) = 12.448, *p* < 0.001], but the Aspect mismatch condition only differed from the Morpho-syntactic violation condition, showing a smaller positivity (*p* < 0.05; Aspect vs. Semantics: *p* = 0.292, ns). In the Left Posterior region, there was also an effect of Condition [*F*(2,56) = 7.797, *p* < 0.05], and the Aspect mismatch condition was marginally less positive than the Morpho-syntactic condition (*p* = 0.05; Aspect vs. Semantics: *p* = 0.582, ns). The Right Posterior region again showed a Condition difference [*F*(2,56) = 4.479, *p* < 0.05]: *Post hoc* comparisons showed no significant difference between the Aspect Mismatch and the other two conditions (Semantics: *p* = 0.950, Morpho-syntax: *p* = 0.196, ns); the Morpho-syntactic condition showed a more positive peak compared to the Semantic condition.

On the midline, a Condition (3) by Electrode (5) repeated measures ANOVA rendered a main effect of Condition [*F*(2,56) = 18.353, *p* < 0.001] and a Condition by Electrode interaction [*F*(8,224) = 5.736, *p* < 0.05]. On Fz, there was an effect of Condition [*F*(2,56) = 18.897, *p* < 0.001]: aspect processing showed a smaller positivity compared to Morpho-syntactic processing (*p* < 0.05) but not compared to Semantic processing (*p* = 0.90). On FCz, the omnibus ANOVA displayed a Condition main effect [*F*(2,56) = 27.920, *p* < 0.001], and again Aspect processing was less positive than Morpho-syntactic processing (*p* < 0.001). On Cz, there were Condition differences [*F*(2,56) = 13.157, *p* < 0.001]: aspect processing was marginally more positive than Semantic processing (*p* = 0.051) but less positive than Morpho-syntactic processing (*p* < 0.05). Looking at posterior electrodes on the Midline, there was a significant overall Condition effect on Pz [*F*(2,56) = 11.093, *p* < 0.001]: aspect processing did not differ from Semantic processing (*p* = 0.798, ns), but showed a smaller positivity compared to Morpho-syntactic processing (*p* < 0.05). On Oz, there was no effect of Condition [*F*(2,56) = 1.879, *p* = 0.171, ns].

Overall, in the P600 time window, aspect mismatches rendered a less positive P600 compared to morpho-syntactic violations, with the latter condition displaying a large P600 effect that was widespread across the scalp. Aspect mismatches were only marginally more positive than semantic violations (localized in the Left Anterior Region and on electrode Cz) and only to some extent was the aspect-related P600 similarly positive compared to the processing of morpho-syntax (Left Posterior Region).

##### Early Negativity time window (250–350 ms)

**FIGURE 5:**
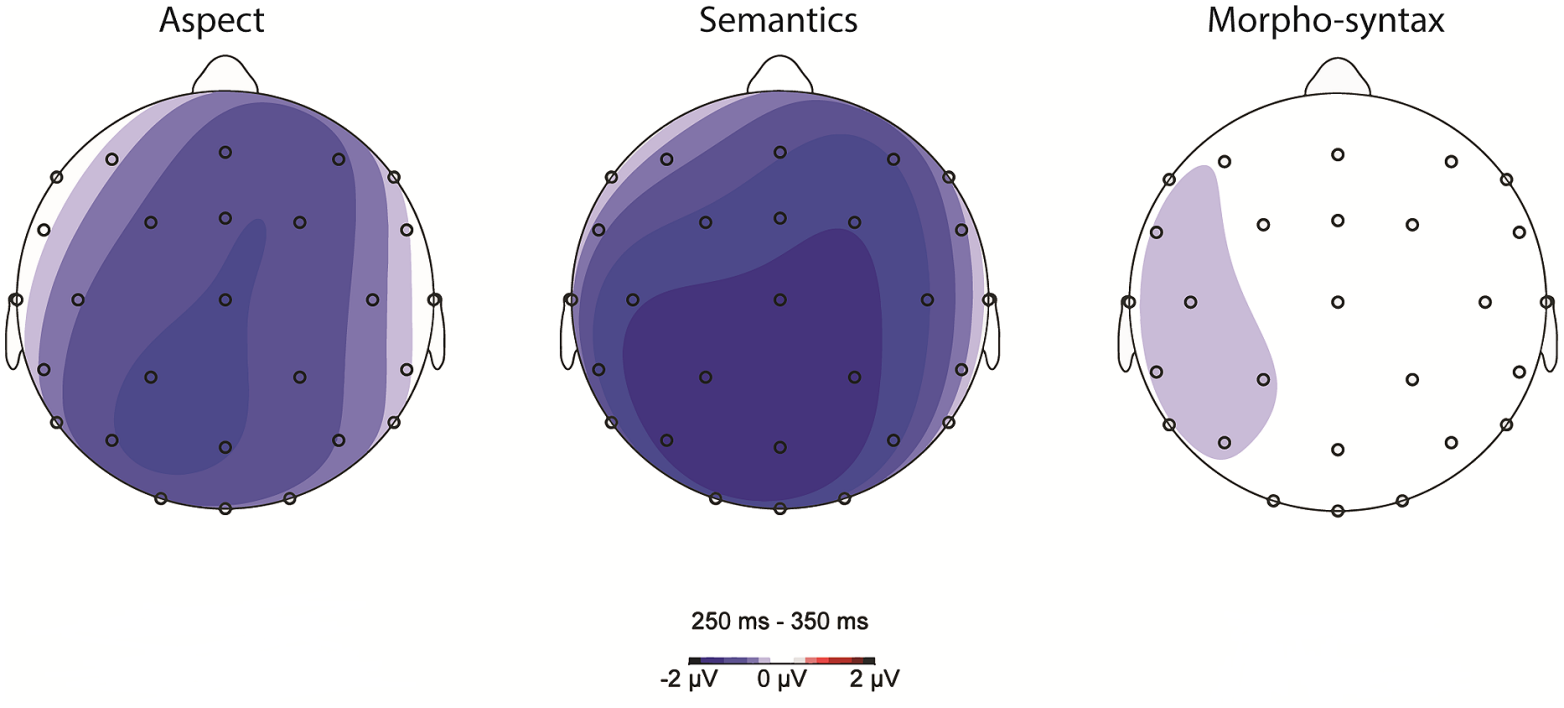
**Scalp topography of aspect, semantic, and morpho-syntactic processing (violation condition minus control condition) between 250 and 350 ms**.

Visual inspection of scalp topographies and grand averaged waveforms showed a short negative peak for the Aspect mismatch and the Semantic violation conditions, between 250 and 350 ms, similar to LAN/Early Negativity effects for tense agreement violations (Steinhauer and Ullman, 2002; Baggio, 2008). **Figure 5** shows the scalp distribution of aspect, semantic, and morpho-syntactic processing (subtracting the control condition from each critical condition) in this time window.

For the lateral electrodes, a Condition (3) by Anteriority (2) and Hemisphere (2) repeated measures ANOVA showed a main effect of Condition [*F*(2,56) = 3.264, *p* < 0.05], and a trend for a three way interaction between Condition, Anteriority, and Hemisphere [*F*(2,56) = 2.792, *p* = 0.069]. Based on our *a priori* interest in the scalp topography of Aspect processing, we ran Condition analyses with a focus on the Aspect mismatch condition for each Region separately. Starting with the Left Anterior Region, a repeated measures ANOVA showed no effect of Condition [*F*(2,56) = 1.058, *p* = 0.351, ns]. In the Right Anterior Region, there was a significant Condition effect [*F*(2,56) = 3.369, *p* < 0.05]: the Aspect mismatch condition did not differ from the Semantic Violation condition (*p* = 0.470, ns), but it was more negative than the Morpho-syntactic violation condition (*p* < 0.05) in this region. In the Left Posterior Region, there was only a trend for an effect of Condition [*F*(2,56) = 2.833, *p* = 0.067], similar to the results obtained for the Right Posterior region [*F*(2,56) = 2.640, *p* = 0.080].

On the midline, we performed a Condition (3) by Electrode (5) repeated measures ANOVA, which showed a main effect of Condition [*F*(2,56) = 5.598, *p* < 0.05] and no Condition by Electrode interaction [*F*(8,224) = 0.379, *p* = 0.931, ns]. Comparing average amplitudes in the Aspect Mismatch condition to the Semantic Violation condition showed no difference [*F*(1,28) = 0.815, *p* = 0.374, ns], but the Aspect mismatch condition was significantly more negative than the Morpho-syntactic condition [*F*(1,28) = 6.368, *p* < 0.05] overall across the Midline.

Between 250 and 350 ms, the aspect condition showed a Negativity. This was also present for semantic violations, but there was no such short-lived negative peak for morpho-syntactic processing. The aspect-related Early Negativity was strongest overall across the Midline of the scalp with a moderate Right Anterior focus.

### Behavioral Data: Off-line Tests

Additional data were collected in several off-line tasks. First, in order to confirm high English proficiency, participants completed the LexTALE test (Lemhöfer and Broersma, 2012). The mean score was 93.89 (SD 5.59)^1^ with low variability in the sample, so, as expected, our participants can be classified as upper advanced users of English (C1 level, according to the CEFR).

Second, typicality ratings were collected. These ratings concerned the semantic content of items in the control condition and those in the semantic violation condition. Participants rated items in the control condition on scale from 1 (not typical at all) to 5 (very typical) with an average rating of 4.83 (SD 0.32), whereas items in the semantic violation condition were given an average rating of 1.90 (SD 0.37). The difference in ratings was significant, as evidenced by a repeated measures ANOVA [*F*(1,38) = 603.863, *p* < 0.001], confirming the intended semantic violation manipulation of the items.

Finally, grammaticality judgements were obtained for morpho-syntactic violations, control, and aspect mismatch sentences. Participants rated sentences from each condition on a scale from 1 (not grammatical at all) to 5 (grammatical). A repeated measures ANOVA comparing mean ratings in the three conditions (correct, aspect mismatch, morpho-syntactic violation) showed a main effect of condition [*F*(2,56) = 222.057, *p* < 0.001]: participants judged morpho-syntactic violations (e.g., *Every Friday, James drink in the bar*; mean judgment = 1.59, *SD* = 0.63) as less grammatical than control sentences (mean judgment = 4.90, *SD* = 0.13), and as less grammatical than aspect mismatch sentences (e.g., *Every holiday, the teacher is climbing in the mountains*; mean judgment = 3.73, *SD* = 0.89; both comparisons *p* < 0.001), confirming the intended morpho-syntactic violation condition. Aspect mismatches, in turn, were rated as significantly less grammatical than control sentences but more grammatical than morpho-syntactic violations (both comparisons *p* < 0.001), reflecting an ‘in between’ pattern concerning grammaticality status. Aspect mismatch sentences were thus not considered to be highly ungrammatical.

## Discussion

The present study explored brain processing of grammatical aspect. We investigated temporal-aspectual agreement relations between morpho-syntactic markers on the verb (progressive *–ing* or simple present tense morphemes) and the preceding temporal context (adverbials in a context sentence). Event-related potentials for a group of English participants to aspect mismatch items (*Right now, Sophie ^∗^swims*) were compared to those evoked by semantic violations and morpho-syntactic violations on the verb phrase. Given that progressive aspect entails a marked morpho-syntactic form and conveys meaning (‘ongoingness’), the aim was to disentangle potential similarities and/or differences with semantic and morpho-syntactic processing by comparing the three violation conditions in the N400 and P600 time windows. In addition, an early time window (250–350 ms) was selected for analysis, based on findings for agreement and tense processing reporting LAN/Early Negativities in this range.

**FIGURE 6:**
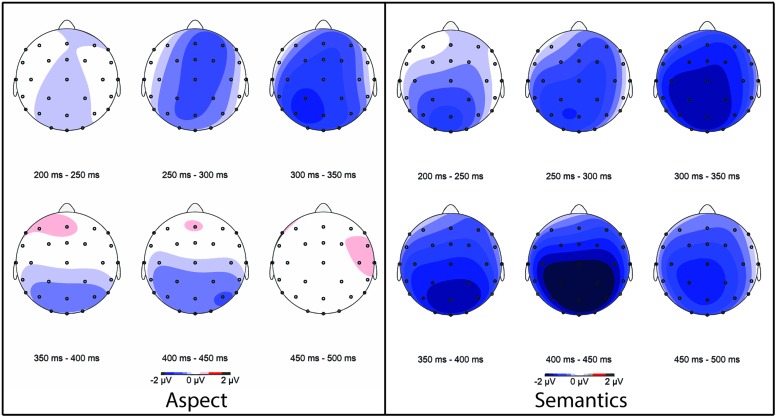
**Scalp topography of aspect processing (left) and semantic processing (right) for every 50 ms between 200 and 500 ms**.

Aspect processing rendered a short-lived Early Negativity (250–350 ms), which neither continued into the N400 time window, nor was followed by a P600. Clear and typical N400 and P600 effects were obtained for semantic and morpho-syntactic processing, respectively. There was no evidence for a biphasic LAN-P600 response in relation to morpho-syntactic processing (similar to Hagoort and Brown, 2000 and Kaan et al., 2000, also looking at number agreement violations). In addition, the semantic violation condition also showed an enhanced negativity in the 250–350 ms time window, with a similar latency but a slightly differing scalp distribution, compared to the aspect mismatch condition (**Figure 6**). **Figure 6** below shows topographic maps for every 50 ms between 200 and 500 ms, further highlighting the distinct patterns of aspect and semantic processing in the Early Negativity and the N400 time windows.

The semantic Early Negativity had its onset in posterior regions before spreading across the scalp, maintaining a strong posterior focus throughout the N400 time window, as is typical for a semantic N400 effect (review in Kutas and Federmeier, 2011). In fact, it is difficult to disentangle the Early Negativity from the N400 on the basis of topography in relation to semantic processing. The aspect-related Early Negativity, however, had a short-lived lateralization in the right anterior region and the midline at the onset of the time window (250–300 ms), before extending across the midline to posterior regions (300–350 ms). In the subsequent N400 range there was no enhanced negativity in relation to aspect.

Focusing on aspect, the overall topography of the Early Negativity is not typical of the LAN usually associated with grammatical agreement processing (review in Molinaro et al., 2011), given the lack of a clear (left) anterior scalp topography. Rather, the distribution is more central. This pattern resembles the findings of Molinaro et al. (2008) who obtained a more central Early Negativity (a more ‘N400-like’ LAN) for gender agreement as compared to phonotactic agreement, the latter of which showed a more typical left anterior distribution. The authors argue that gender agreement is to some extent reliant on non-syntactic lexical information, i.e., the specific word forms involved, and not limited to the processing of morpho-syntactic features alone which would render an Early Negativity with the typical left anterior topography, i.e., the LAN obtained for phonotactic agreement processing (cf. Molinaro et al., 2008); the central distribution may thus reflect the involvement of information at the lexical level. The present centrally distributed aspect-related Early Negativity suggests that the lexical properties of the verb are relevant for aspect agreement checking: Aspectually marked (Sophie *is swimming)* and unmarked verbs (Sophie *swims*) in English differ considerably at the lexical level, given that verb phrases marked with progressive aspect always start with the same form, a finite copula (e.g., *is* V-ing).^2^ In line with Molinaro et al. (2008), we argue that the reader may have created expectations regarding the specific verb form on the basis of the temporal information in the preceding context sentence, which functions as a trigger for temporal agreement checking. In the present experiment, these expectations based on temporal information either did or did not involve the highly specific form of the copula ‘*is,’* reflecting a progressive or non-progressive perspective on the activity. A violation of these expectations (“is” or “no is”) upon encountering the verb phrase may be reflected in the short-lived Early Negativity.

Our findings for aspect processing also bear some similarity to Early Negativities reported in the domain of auditory sentence processing: Studies have reported a negativity which precedes the N400 and which is brought about by a mismatch of an encountered form with (pre)lexical phonological expectations (Connolly and Phillips, 1994; Newman et al., 2003) or semantic expectations (van den Brink et al., 2001). In these studies, participants’ expectations were strongly biased toward (part of) this specific word form given a preceding context. In Connolly and Phillips (1994), a frontal-temporal negativity (Phonological Mismatch Negativity, PMN between 270 and 300 ms) was elicited when there was a mismatch regarding an expected phoneme, but no semantic violation on a word (e.g., “The pig wallowed in the pen [expected: mud]”). The PMN was not followed by an N400. In another study by van den Brink et al. (2001), it was argued that a similar Early Negativity effect (labeled the N200) stemmed from a mismatch between the perceived word and top-down contextually activated semantic and syntactic features of the word. In their interpretation, semantic information did play a role for the elicitation of the Early Negativity. In both cases, the Early Negativities were driven by expectations at the prelexical or lexical level and this is in line with our functional interpretation of the aspect-related Early Negativity outlined above. It is important to note, however, that there is evidence that the PMN and N200 are exclusive to the auditory domain (Connolly and Phillips, 1994) so their resemblance to the present findings is speculative. Turning then to visual word recognition processes specifically, one may also draw comparisons to an early negativity (the N250) acclaimed to reflect sublexical or lexical information processing (Holcomb and Grainger, 2006), driven by expectations at the *orthographic* level. However, such effects were obtained in word priming paradigms, again different from the present study targeting sentence processing.

In all, the functional explanation of the LAN in agreement processing is not much different from the expectation-driven basis of both the auditory PMN, the N200 and the visual N250: the LAN marks a violation of expectations concerning a morphological inflection. This inflection is expected on the basis of a ‘trigger’ in the preceding context, which can be a noun, pronoun, determiner, etc. (see Molinaro et al., 2011). Our interpretation of the aspect-related Early Negativity as the result of a violation of expectations at form level given a biasing (temporal) context would fit all of these expectancy-related lines of reasoning very well. We may even put forward the hypothesis that the presently and previously reported pre-N400 Early Negativities, for different domains of study and different modalities, involve the same type of neural mechanism and are triggered by a violation of form-level expectations. Of course, this hypothesis warrants further research.

Specifically, in the present study the aspect mismatch condition elicited only an Early Negativity, but not an additional N400 (nor a P600), supporting the (pre)lexical and thus *orthographic* nature of the expectations generated by the context. In this sense there is a specific resemblance to the condition in Connolly and Phillips (1994) which elicited *only* a PMN, i.e., the semantically appropriate word containing an unexpected initial phoneme (note again that their findings were obtained in the auditory domain), suggesting that our finding of only an Early Negativity for aspect processing relates to expectations at the form level. Interestingly, however, the semantic condition in the present study did elicit both an Early Negativity and an N400 (see discussion below).

The aspect mismatch condition did not lead to a clear P600 effect (though there is a marginal tendency in the data, see above), even though the biphasic Early Negativity (or LAN) plus P600 pattern is frequently reported for grammatical agreement. The P600 of agreement processing is often interpreted as an attempt at reintegrating the unexpected word form with the preceding context (Molinaro et al., 2011). Given the lack of an aspect-related P600, for violations of aspect agreement this process may either not be reflected in the P600 (i.e., it happens very rapidly before this stage), or there is no such reanalysis. With regard to our participants’ behavior in the *post hoc* grammaticality judgment task, we see that the majority of them in fact did *not* overtly judge aspect mismatch sentences as ungrammatical. On the other hand, participants judged morpho-syntactic violation sentences, e.g., *Sophie ^∗^are swimming*, to be ungrammatical, and here we did find a robust P600 modulation. The lack of a P600 for aspect suggests that participants did not need to reintegrate the unexpected aspect marker with the context (e.g., *swims*, when one was expecting *is swimming* after *Right now*), as they generally accepted the mismatch sentences as grammatical, and this is reflected in their brain potentials. The Early Negativity effect, on the other hand, resembles the early and almost automatic detection of a violation of the expected verb form (see above), an early stage of processing which, arguably, is not sensitive to overt grammaticality judgements.

The question arises why our participants overall accepted aspect mismatch sentences as grammatical; this type of violation is formally considered to be ungrammatical (following the average English language text book). Arguably, this higher tolerance toward aspect mismatches might be a consequence of frequent exposure to non-native English language use, which holds true for our specific sample of native English speakers who were residing in a foreign country at the time of testing. They were, however, still using English as their primary language of communication. In second language production of English (and more generally), overuse or avoidance of grammatical aspect is a common pattern (see, e.g., Bardovi-Harlig, 2000; Salaberry and Shirai, 2002; Montrul and Slabakova, 2003; Flecken, 2011; Roberts and Liszka, 2013) because of the difficulty in learning to use the forms appropriately given their strong dependence on context (e.g., *I am working at university for a living* would be a common overextension of the progressive to habitual contexts). High grammaticality judgements of aspect mismatch sentences could thus be the result of frequent exposure to this type of formally inappropriate use of aspect. Moreover, it is also the case that in specific contexts of language use, the ongoing – habitual distinction regarding the English progressive is not adhered to strictly (e.g., in sports coverage, *Right now*, *Robben passes the ball and he scores*). Follow-up studies targeting different populations may shed more light on this issue.

How then can we explain an effect in the pre-N400 Early Negativity time window (250–350 ms) for semantic processing? Close inspection of scalp topographies shows that the pattern could resemble an early onset of the N400, suggesting that the semantic properties of the verbs were retrieved rapidly, potentially also driven by the strongly biasing preceding context. Remember that the context contained temporal information as well as information relevant to the semantics of the upcoming verb, i.e., the location at which an activity typically takes place: *What is Sophie doing in the pool, right now?*. The reference to the location could bias expectations to the specific action verb and its form: all action verbs were highly frequent (e.g., *to swim, to cook, to walk, to dance*), making it likely that the location reference *in the pool* would immediately prime or drive expectations toward the specific verb form *swim*. In this sense, then, the semantic Early Negativity reflects a violation of the readers’ expectations concerning the lexical properties of the verb based on semantic information, i.e., following *in the pool* one would immediately expect the specific word *swim* rather than *cook* (verb X rather than verb Y). In this interpretation, the aspect-related Early Negativity, on the other hand, would reflect expectations at the level of the verbal inflection based on preceding temporal information (following *right now* one would expect the form *is X-ing* rather than *X-s*).

As discussed above, previous work on aspect processing tested aspect violations that were locally morpho-syntactically erroneous (at the level of the verb phrase, e.g., a verb with *both* a perfective and a progressive aspectual morpheme, Zhang and Zhang, 2008), rather than violations of aspect *agreement* between context and verbal marking, as is the case at present. Zhang and Zhang (2008) showed an Early Negativity followed by a P600. It is likely that the local morpho-syntactic violation lead to the P600, reflecting repair or reintegration processes (see discussion in Baggio, 2008). Specific tense violations (e.g., *Yesterday, I ^∗^sail Diane’s boat to Boston*) also resulted in a LAN/Early Negativity response (Steinhauer and Ullman, 2002; Baggio, 2008). Crucially, those tense violations were also violations of temporal agreement, brought about by a mismatch of preceding temporal adverbials with tense marking on the verb. In Steinhauer and Ullman (2002), however, the Early Negativity *was* followed by a P600. The discrepancy between our findings and their P600 could be caused by methodological differences regarding either the type of violation concerned (tense or aspect) and/or the type of population tested (native English participants in an English-dominant country or native English speakers in a country with a different dominant language). Future studies should address these issues.

## Conclusion

We find an aspect-related Early Negativity (250–350 ms) in the ERPs of English native speakers in relation to aspect mismatch sentences (*Right now, Sophie ?swims in the pool*). Aspect processing evoked neither the N400, nor the P600, which were obtained, respectively, for semantic violations (*Right now, Sophie ^∗^is cooking in the pool*) and morpho-syntactic violations (*Right now, Sophie ^∗^are swimming in the pool*) in the same participants. The differences in ERPs between the three conditions suggest that aspect processing reflects operations that are neither purely semantic nor exclusively morpho-syntactic in nature.

Instead, we argue for viewing the processing of aspectual relations as an agreement operation. The temporal information encoded in aspectual morphology should be in agreement with the temporal frame of reference set up by the context (e.g., adverbials like *Right now*, or other verbal aspect markers in the preceding discourse). The preceding temporal information leads to expectations regarding the form of the verb phrase (*Sophie is X-ing* or *Sophie X-s*). The Early Negativity in response to mismatch items reflects a violation of this expectancy during the temporal agreement checking process. In the present study’s population of English participants, the mismatch did not lead to additional reintegration effort (typically reflected in a P600 modulation for agreement violations). This ties in with their *post hoc* grammaticality judgements, showing overall high acceptance of aspect mismatches.

We propose that aspectual morphology establishes agreement relations with other markers of temporality in the context. The temporal agreement checking between this contextual information and verbal aspect marking occurs rapidly (within 350 ms). In all, temporal-aspectual information, a fundamental part of communicating about events, forms a unique contribution to online sentence processing.

## Conflict of Interest Statement

The authors declare that the research was conducted in the absence of any commercial or financial relationships that could be construed as a potential conflict of interest.

## Footnotes

1 LexTALE scores reflect the percentage of correct responses, corrected for the unequal proportion of words and non-words in the test, by averaging the percentages correct for the two item types: [(number of words correct/40^∗^100) + (number of non-words correct/20^∗^100)]/2 (www.lextale.com).

2 Note that we controlled for different lengths of verb phrases (progressive – long and non-progressive – short) in all conditions and found no effect in relation to this.
