# Supplementary material for: ‘Right Now, Sophie ∗Swims in the Pool?!’: Brain Potentials of Grammatical Aspect Processing
Source: Front Psychol. 2015 Nov 23;6:1764. doi: 10.3389/fpsyg.2015.01764 (PMC4655232; doi:10.3389/fpsyg.2015.01764)
Supplement: Supplementary file 1 [file Data_Sheet_1.DOCX]

**Appendix**

List of items in the reading task (verb + PP combinations in control and semantic violation conditions)

| Item | Control  condition | Semantic violation condition | PP (location) |
| --- | --- | --- | --- |
| 1 | swim | cook | in the pool |
| 2 | climb | study | in the mountains |
| 3 | cook | swim | in the kitchen |
| 4 | dance | read | in the club |
| 5 | read | dance | in the library |
| 6 | work | drink | in the office |
| 7 | surf | work | in the ocean |
| 8 | paint | shower | in the studio |
| 9 | hike | eat | in the forest |
| 10 | drink | surf | in the bar |
| 11 | shower | paint | at the laundromat |
| 12 | study | walk | at school |
| 13 | eat | climb | in the restaurant |
| 14 | bathe | drive | in the bathroom |
| 15 | shop | sail | in the mall |
| 16 | play | hike | at the playground |
| 17 | drive | exercise | on the road |
| 18 | sail | shop | on the water |
| 19 | exercise | sing | at the gym |
| 20 | sing | bathe | on stage |
